# Fetal Malnutrition and Associated Factors among Term Newborn Babies at Birth in South Gondar Zone Hospitals, Northwest Ethiopia

**DOI:** 10.1155/2021/5005365

**Published:** 2021-11-28

**Authors:** Desalegn Tesfa, Fentaw Teshome, Birhanie Ambaw

**Affiliations:** Department of Public Health, College of Health Sciences, Debre Tabor University, Debre Tabor, Ethiopia

## Abstract

**Background:**

Undernutrition contributes to almost half of all under-five deaths. Fetal malnutrition (FM) has been implicated in both short- and long-term consequences among fetal, neonatal, and adult life. Currently, Ethiopia lacks information on the prevalence of fetal malnutrition and its associated factors. This study, therefore, is aimed at assessing the prevalence of FM at birth and its associated factors in South Gondar zone hospitals, northwest Ethiopia.

**Methods:**

A cross-sectional study was carried out from November 1, 2019, to April 30, 2020, among four hospitals of South Gondar zone. All women with their live newborns who met the eligibility criteria were included. Clinical assessment of nutritional status including other anthropometric measurements was done immediately after delivery. The newborn was declared as fetal malnourished if the clinical assessment of nutritional status cut-off point is less than 25. Data were collected by trained clinical midwives. Besides bivariate regression analysis, a multivariable logistic regression analysis was done to identify associations.

**Results:**

A total of 1592 mothers with their live newborns participated in this study. The prevalence of fetal malnutrition was 21.7% (95% CI: 19.7-23.9). Intimate partner violence (AOR: 1.97, 95% CI: 1.52-2.56), placental weight less than 512 grams (AOR: 2.76, 95% CI: 2.13-3.57), and small for gestational age (AOR: 1.96, 95% CI: 1.46-2.62) were significantly associated with fetal malnutrition.

**Conclusions:**

The prevalence of fetal malnutrition was a public health problem in this study. Intimate partner violence, placental weight, and small for gestational age were found the most significant variables. To avert fetal malnutrition, positive family relation and additional or balanced nutritional supplementation during pregnancy are critical. We recommend researchers do clinical follow-up research which comprises a detailed investigation of placental, maternal, and fetal factors including genes.

## 1. Background

Fetal malnutrition (FM) is defined as intrauterine loss or failure to acquire a normal amount of subcutaneous fat and muscles [1]. Fetal malnutrition, small for gestational age, and intrauterine growth restriction are not synonymous; one may occur without the others [2–4]. An infant who is classified with intrauterine growth restriction may, or may not, also be classified as small for gestational age. Likewise, an infant who is classified with intrauterine growth restriction (IUGR) and/or small for gestational age may, or may not, have fetal malnutrition [2, 3]. Fetal malnutrition represents a situation that may exist at almost any birth weight, and it is independent of birth weight and gestational age [4–7]. Some intrauterine growth restrictions or small for gestational age babies may not be malnourished [7]. Fetal malnutrition adversely affects body composition, metabolism, and enzymatic functions [1, 4]. If the baby is malnourished, tissue structure and composition, metabolic reactions, and enzyme functions in many systems are affected. Among newborns, the features of fetal malnutrition can be clinically recognized by the observation of several morphological changes in the subcutaneous fat and muscle tissues, skin, and hair [4, 8]. Fetal length, head circumference, and weight are significantly reduced if the malnutrition occurred in the second trimester, whereas if both of them are in the normal range and weight is significantly less than the gestational age, an insufficient nutritional supply occurred during the late third trimester [4].

Perinatal complications and/or central nervous system sequelae occurred primarily among malnourished babies whether of appropriate for gestational age (AGA) or small for gestational age (SGA), but not those who were simply SGA [4, 6, 9]. Pneumonia, diarrhea, and malaria remain the leading causes of child death globally, claiming the lives of around 6000 children under five each day. Undernutrition contributes to almost half of all under-five deaths [10]. Fetal malnutrition has been implicated in both short- and long-term adverse outcomes. Globally, around 20 million babies are born per year with birth weights less than 2500 g, and around one out of three to two out of five of these infants are born at term gestation (gestational age between 37 and 42 weeks) and therefore are SGA. Thus, at least one-half of these (about 3 million) babies are fetally malnourished [4].

The incidence of fetal malnutrition among newborns using clinical assessments of nutritional status (CAN score) was 84.2% among small for gestational age babies, 12.9% among appropriate for gestational age babies [6], and 54.8% among preterm infants [7]. With the same measurement, the prevalence of fetal malnutrition was 16.7% to 19% [5, 7, 11, 12]. Of the 19.6% of malnourished babies, two-fifth of them had IUGR, 59.9% were appropriate for gestational age, and 1.9% were SGA [7]. Fetal malnutrition has been implicated in both short- and long-term consequences among children and adults, characterized by an increased risk of cardiovascular diseases and insulin resistance [4, 6, 12, 13]. The incidences of perinatal hypoxia including related morbidities and late neurodevelopmental problems have been reported to be higher among term infants with fetal malnutrition [2, 4, 14]. Psychometric studies revealed that children with fetal malnutrition were more likely to have lower Intelligence Quotient (IQ) scores, required special education, intellectual disorder, and learning disability among late childhood compared to children without fetal malnutrition [2, 9, 13].

Risk factors for FM included maternal factors such as advanced maternal age, parity, race, low educational status, poor wealth status, anemia, malaria, and chronic disease (like pregnancy-induced hypertension and diabetes mellitus) [1, 8, 12, 15]. Even though different experts identified fetal malnutrition at birth by using various assessment methods, clinical assessment of nutritional status (CAN) score, a simple clinical index for identifying the term fetal malnutrition, is a good indicator than other anthropometric approaches [5, 9, 16, 17]. But for a preterm infant, body mass index (BMI) and ponderal index (PI) are simple and easy tools to use in assessing fetal malnutrition [18]. Clinical assessment of nutritional status is also important for the prediction of neonatal mortality associated with fetal malnutrition [19]. The thought of FM was initially developed by Clifford [4] and was defined by Scott and Usher, and it can be clinically assessed by physical assessment using the clinical assessment of nutritional status (CAN) score [5, 14].

Several complications during childhood are associated with the nutritional status of infants at birth [18, 20]. Therefore, the nutritional status of newborns must be evaluated properly after birth. Assessment of fetal malnutrition by physical indices is simple, inexpensive, and does not need advanced medical equipment [5, 12, 13, 21]. Currently, Ethiopia lacks information on FM's prevalence and its associated factors at birth. This study is aimed at assessing the prevalence of FM at birth and its associated factors by using physical indices (nine superficial readily detectable signs of malnutrition). Detection of fetal malnutrition at birth is thus important for identifying those newborns who are at higher risk, and it is useful for the government to design strategies, therefore decreasing intrauterine fetal malnutrition. Subsequently, it is very imperative to achieve the sustainable development goal of child health particularly in Ethiopia where child mortality is high.

## 2. Methods

### 2.1. Study Setting

This study was carried out among the four hospitals of the South Gondar zone (Debre Tabor, Nefas-Mewicha, Mekane Eyesus, and Addis Zemene). South Gondar is a zone in Ethiopian Amhara region [22]. In Ethiopia, even though there is a substantial increment in institutional delivery from 5% in 2005 to 48% in 2019, still greater than half (52%) of the women have delivered at home [23]. Based on the 2019 South Gondar zone report, this zone has a total population of 2,609,824. Of these, around 527,967 reproductive age group females and 87,955 pregnant women were found in the zone.

The zone has 8 hospitals, 96 health centers, 394 health posts, and 118 private health institutions. All of the hospitals give similar services except Debre Tabor General Hospital that serves as a referral center for private and governmental institutions. Averagely, 138, 336, 122, and 133 deliveries were conducted in two months among Nefas-Mewicha, Debre Tabor, Mekane Eyesus, and Addis Zemene Hospitals, respectively.

#### 2.1.1. Study Design

Hospital-based cross-sectional study was conducted from November 1, 2019, to April 30, 2020.

#### 2.1.2. Inclusion

In this study, all women with the gestational age of 37-42 weeks and who have given singleton live birth among four hospitals (Nefas-Mewicha, Debre Tabor, Mekane Eyesus, and Addis Zemene hospitals) from November 1, 2019, to April 30, 2020, were included.

#### 2.1.3. Exclusion

Women who could not answer the intended questions because of illness and mental problems, mothers who died because of complications of labor, mothers referred to other higher health institutions, new birth with incomplete placenta, newborns with a congenital abnormality, and those born to mothers with known gestational diabetes mellitus were excluded.

### 2.2. Sample Size Determination

All women who gave live birth from November 1, 2019, to April 30, 2020, among the four hospitals and fulfilled the eligibility criteria were included under this study. Hence, the sample size for the current study consisted of 1592 (Debre Tabor (916), Nefas-Mewicha (241), Addis Zemene (211), and Mekane-Eyesus (224)) mothers with their live singleton newborns.

*Outcome (dependent) variable* is fetal malnutrition.

*Independent variables* are sociodemographic characteristics of the mothers and husband (intimate partner), newborn characteristics, and maternal obstetric factors.

### 2.3. Operational Definitions

*Clinical assessment of nutrition status* (*CAN score*) is a scoring system based on nine superficial readily detectable signs of malnutrition among the newborn baby [4, 19].

*Rapid assessment of gestational age at birth* is the gestational ages derived from the total scores of skin texture (4 items), skin color (4 items), breast size (4 items), and ear firmness (4 items) [24]. But for this study, gestational age was estimated by the last menstruation period (LMP) and ultrasound estimation (either of the two), but if two of them (LMP or ultrasound estimation) were impossible, we used the above (rapid assessment of gestational age at birth) estimation method.

*Small for gestational age* (*SGA*) is defined as birth weight less than the 10^th^ percentile for gestational age using weight percentile chart [15].

*Household wealth status* is computed by principal component analysis from different variables such as the presence of own farmland, own toilet facility, bank account, mobile phone, electricity, the roof of the house with corrugated iron sheets, and number of cows/oxen, horses/mules/donkeys, goats/sheep, and chicken [25].

*Chronic disease* is defined as women who had bronchial asthma, heart diseases, hypertensive disorder (gestational and nongestational), diabetes, and chronic renal disease through history or from their client chart.

*Violence* was assessed as exposure to physical violence (6 items) such as slapping, hitting, kicking, and beating; sexual violence (3 items) including forced sexual intercourse and other forms of sexual coercion; and emotional (psychological) abuse (6 items) such as insults, belittling and intimidation, and controlling behaviors (7 items) including isolating a person from family and friends, monitoring their movement, and restricting accesses to financial resources. Women were asked to indicate whether they had experienced any of the violent acts during the current pregnancy and classified based on World Health Organization (WHO) classification [26].

### 2.4. Data Collection Tools and Procedures

Questionnaires were developed after reviewing relevant literature [4, 19, 24] to include all the possible variables that address the objective of this study. Firstly, the questionnaire was developed in English and translated into the local language (Amharic) and finally retranslated into English by a language expert. For each hospital, four clinical midwifery health professionals participated as data collectors. The freshly delivered placenta was cleaned from blood clots and then weighed by using an infant weighing scale to the nearest gram. For this study, we used the mean of the placental weight to classify and the mean was 512 grams. Placenta included membranes and umbilical cord and was cut approximately 5 cm from the neonate side. Detailed examination of each baby was carried out by data collectors.

Anthropometric measurements were carried out on babies using standard methods as soon as possible after birth and within 24 hours. The nutritional status of babies was assessed using Metcoff's clinical assessment of nutritional status score (CAN score) chart [4, 13]. The chart contains scores for nine superficial and readily detectable signs of FM. Scoring was done based on inspection and hands-on estimation of the degree of loss of subcutaneous tissue and muscles. For each point of assessment, the degree of loss of subcutaneous fat was scored by applying a maximum score of four for no evidence of malnutrition and the lowest score of one for the worst evidence of malnutrition. The highest attainable score was 36 and the lowest was 9. Babies with a total score below 25 were classified as having FM [13].

### 2.5. Data Quality Assurance

To ensure the quality of data, training was given for all data collectors at each hospital for one day on the overall procedure of data collection by the principal investigator and pediatricians supported with video [22]. The questionnaire was pretested before the actual data collection time on 5% of the sample. Placental weight and weight of the newborn baby were measured immediately after birth and recorded to the nearest decimals. Immediately after delivery, the maternal coagulum was removed from the placenta, and the placenta was placed in a plastic bag to be weighed on a pediatric scale previously calibrated. Detailed examination of each baby was carried out by data collectors. The supervisors closely follow the day-to-day data collection process and ensure the completeness and consistency of the questionnaire administered each day. The collected data were reviewed and checked for completeness before data entry.

### 2.6. Data Processing and Analysis

All the interview questionnaires were checked manually for completeness and consistency before entry. Then, the data were entered, coded, and cleaned using EPI INFO Windows version 7 statistical software. The entered data were exported into the Statistical Package for the Social Sciences (SPSS) version 20 for further analysis. Descriptive statistics such as frequency distribution and percentage were performed. Bivariate logistic regression was performed for each independent variable with the outcome variable. All variables whose bivariate test had a *p* value ≤ 0.25 were a candidate to consider into a multivariable logistic regression analysis to avoid confounders. Hosmer and Lemeshow's goodness-of-fit test was done to check the appropriateness of the data for multivariable logistic regression analysis. Crude and adjusted odds ratio together with their corresponding 95% confidence interval were computed to see the strength of association between independent and dependent variables. For all statistical significant tests, *p* value < 0.05 was used as a cut-off point.

## 3. Results

### 3.1. Sociodemographic Characteristics of the Respondents

Of all, 1592 women have participated in this study. Around two-fifth (634, 39.8%) of the mothers were found in the age category of 25-29 and one out of three (33.7%) of the mothers was able to read and write. Almost all (1537, 96.5%) of them were living in union (with their husband). The majority (88.8%) of the mothers lived with a total family size of six or less. One out of five (20.8%) neonates was delivered with a weight less than 2500 grams (low birth weight). The majority (84%) of the mother's height was ≥150 cm (Table 1).

### 3.2. Obstetric Factors

One in five (20.6) of the women's pregnancy intervals was less than 24 months. Of those who had a short interpregnancy interval, 84 (5.3%) had a very short interpregnancy interval (<12 months). Besides, 967 (60.7%) and 297 (18.7%) of the participants had an interpregnancy interval of 24-59 months and more than 60 months, respectively. The majority (91.6%) of the women attended at least one ANC follow-up. Of those who had at least one ANC follow-up, 477 (30%) and 568 (35.7%) of the participants have attained for third and fourth times, respectively. Around two-fifth of their placental weight was below 512 grams. Greater than one-third (35.7%) of the mothers were exposed to intimate partner violence (Table 2).

### 3.3. Factors Associated with Fetal Malnutrition

In bivariate logistic regression newborn weight, intimate partner violence, gestational age, placental weight, and altitude were significantly associated with fetal malnutrition. However, in multivariable logistic regression, intimate partner violence, gestational age, and placental weight were significantly associated with fetal malnutrition. Accordingly, the odds of having fetal malnutrition were 2 times higher among mothers who were exposed to intimate partner violence compared to mothers who did not encounter intimate partner violence (AOR: 1.97, 95% CI: 1.52-2.56). Newborns delivered at less than the 10^th^ percentile (SGA) were 2 times more likely exposed to fetal malnutrition than those born normal for its gestational age (AOR: 1.96, 95% CI: 1.46-2.62), and fetuses delivered at a placental weight less than 512 grams were 3 times more likely exposed to FM than those delivered at a placental weight less than 512 grams (AOR: 2.76, 95% CI: 2.13-3.57) (Table 3).

## 4. Discussion

Clinical evidence of wasting in a newborn, known as fetal malnutrition (FM), is regarded as an index of intrauterine difficulties in the baby [27]. Accurate assessment of the nutritional status of the fetus has been a major concern to many clinicians because of the potentially serious sequelae of malnutrition on multiple organ systems [14]. In this study, therefore, we tried to assess the prevalence of fetal malnutrition and its associated factors among term newborn babies at birth in South Gondar zone hospitals using the clinical assessment of nutritional status based on nine superficial readily detectable signs of malnutrition. The result of this study revealed that the prevalence of fetal malnutrition in South Gondar zone hospitals was 21.7% (95% CI: 19.7-23.9).

This study was consistent with other studies conducted in Nigeria (18.8%) [12] and in India (17.5% [6], 19.6% [7], 24% [13], and 21.2% [16]) which was carried out in the same country by different investigators at different periods. The similarity of our study with these studies might be the target population. Because the current and the above studies used the same target population (term newborn), the same fetal malnutrition screening tool (CAN score), and the same cut-off point to determine fetal malnutrition, other fetal malnutrition screening methods such as intrauterine growth curves or 10^th^ percentile of birth weights were missed about 10% of appropriate for gestational age cases of malnourished neonates [7]. Likewise, using clinical assessment of nutritional status scores as assessment methods can suspend 12.5% of appropriate for gestational age that classified wrongly as malnourished by intrauterine growth curves or 10^th^ percentile of birth weight measurements [7, 14]. Thus, it is obvious that the CAN score was able to diagnose more precisely cases of malnourished babies at term [4, 7, 14]. Nevertheless, this study was lower than studies conducted in New Delhi, India (40%) [19], in Hyderabad, India (49.6%) [28], and in Turkey (54.8%) [6]. The low prevalence of our study to New Delhi, India, might be the target population and their period (data collection, year), because in India they include preterm neonates (delivered with less than 37 weeks of gestation) and the time was so far compared to the current study. Similarly, the economic status and the health-seeking behaviors of the population during pregnancy changed over time. And the high prevalence in Hyderabad, India, was due to the cut-off point they used to identify fetal malnutrition (they used clinical assessment of nutritional status score cut-off ≤ 24), but our study used 25 as a cut-off point like most of the other studies. The present study was, however, higher than studies conducted in Nigeria (16.7%) [11] and in Bhopal, India (8.3%) [29]. The discrepancy between the current study and the study done in Nigeria might be that, in Nigeria, the participants were mothers who received antenatal care and delivered in the tertiary hospital. Therefore, it is obvious that those mothers who attend antenatal care follow-up had not only lower fetal malnutrition but also lower intrauterine growth retardation, small for gestational age, and low birth weight, because while the mothers attend their antenatal care, the healthcare providers advise them about nutrition, weight gain, screening of infection, and iron with folic acid supplementation. In the current study, intimate partner violence was one of the significant factors for fetal malnutrition (AOR: 1.97, 95% CI: 1.52-2.56) and it was in line with other studies that showed intimate partner violence was a significant predictor for adverse pregnancy outcome (AOR: 5.34; 95% CI: 1.97-14.46) [30], because it is a fact that if the mothers are exposed to intimate partner violence, they did not give special attention to their pregnancy like preconception care and antenatal care follow-up, and if they are exposed to intimate partner violence, they were not taking additional and balanced nutrition during their pregnancy. It was also supported with other studies: intimate partner violence was associated with over five times increased odds for any adverse neonatal outcome (small for gestational age, low birth weight, and preterm babies; AOR: 5.34, 95% CI: 1.97–14.46) and specifically with a fourfold exposed to small for gestational age (AOR: 4.00, 95% CI: 1.58–9.97) [31]. An infant born with small placental weight was 3 times more likely to experience FM (AOR: 2.76, 95% CI: 2.13-3.57), and it was supported by a study done in Finland [32]. Since the fetus is not a parasite, it depends on the extraction and nutrient composition of the umbilical blood and the flow rate and the capacity to utilize the extracted nutrients. Subsequently, a small placenta has thinner umbilical cords which lead to uteroplacental insufficiency to feed the fetus and the fetus is exposed to fetal malnutrition, because placental inadequacy and insufficient perfusions of the fetus might result in fetal suffering and premature initiation of the hypothalamus-pituitary-adrenal axis [33]. Gestational age was also the significantly associated factor (AOR: 1.96, 95% CI: 1.46-2.62) for fetal malnutrition. Being SGA is a significant factor for fetal malnutrition, and it was supported by studies conducted in Royal Victoria and McGill, USA, and in Oklahoma [1, 4, 6, 7].

## 5. Conclusions

To our knowledge, even though this study was the first study in the country, the prevalence of fetal malnutrition was a major public health concern. Intimate partner violence, placental weight less than 512 grams, and small for gestational age were found to be the most significant variables. To avert fetal malnutrition, effort in raising sensitization on intimate partner violence and its consequence through health extension workers and health professionals and screening, counseling, treating, and follow-up of abused women are needed. Primary prevention at the perinatal period by involving the couple decreases the risk of FM related to intimate partner violence. Increasing appropriate for gestational age and placental weight by additional and/or balanced nutritional supplementation during pregnancy is critical. We recommend researchers do further research which comprises a detailed investigation of placental, maternal, and fetal factors including genes.

### 5.1. Limitations

For this study, there might be a recall and social desirability bias especially for intimate partner violence and marital status. There might be also gestational age estimation bias because of the recall of the last menstrual period, and there was also a limitation during ultrasound estimation and rapid gestational age assessment. There may be a subjective nature, like all other scoring methods used in the evaluation of neonates.

## Figures and Tables

**Table 1:** Sociodemographic characteristics of the respondents and the newborns in South Gondar zone hospitals, northwest Ethiopia, 2020.

Characteristics	Frequency	Percent
Current maternal age		
≤19	112	7.0
20-24	391	24.6
25-29	634	39.8
30-34	236	14.8
35-39	168	10.6
≥40	51	3.2
Educational status of the mother		
Unable to read and write	437	27.4
Able to read and write	536	33.7
Primary	170	10.7
Secondary	222	13.9
College and above	227	14.3
Marital status		
Currently married	1537	96.5
Single	25	1.6
Divorced	30	1.9
Age of the husband/friend		
<20	22	1.4
20-24	228	14.3
25-29	331	20.7
30-34	326	20.5
35-39	364	22.9
≥40	321	20.2
Husband/intimate partner educational status		
Unable to read and write	339	21.3
Able to read and write	415	26.1
Primary	150	9.4
Secondary	386	24.2
College and above	302	19.0
Total family size		
1-3	882	55.4
4-6	532	33.4
≥7	178	11.2
Wealth quintile		
Very poor	80	5.0
Poor	315	19.8
Middle	818	51.4
Better	379	23.8
Altitude		
<2000 m	426	26.8
2000-3000 m	298	18.7
>3000 m	868	54.5
Sex of the newborn		
Male	690	43.3
Female	902	56.7
Weight of the newborn		
<2500 grams	331	20.8
≥2500 grams	1261	79.2
Gestational age classification		
Small for gestational age	328	20.6
Appropriate for gestational age	1264	79.4
Have known chronic disease		
No	1505	94.5
Yes	87	5.5
Intimate partner violence		
Yes	569	35.7
No	1023	64.3

Mean ± SD = 3186 + 446 g.

**Table 2:** Obstetric factors of the mothers in South Gondar zone hospitals, northwest Ethiopia, 2020.

Characteristics	Frequency	Percent	
Pregnancy interval			
<12 months	84	5.3	
12-23 months	244	15.3	
24-59 months	967	60.7	
≥60 months	297	18.7	
Gravidity			
≤2	705	44.3	
3-4	644	40.5	
≥5	243	15.3	
Anemia			
No	1340	84.2	
Yes	252	15.8	
Number of ANC visits			
No visit	134	8.4	
1 visit	126	7.9	
2 visits	287	18.0	
3 visits	477	30.0	
4 visits	568	35.7	
Placental weight			
<512 grams	609	38.3	Mean ± SD = 512 ± 100.2 g
≥512 grams	983	61.7	
Height of mother			
<150 centimeters	255	16.0	Mean ± SD = 166 ± 8.4 cm
≥150 centimeters	1337	84.0	

**Table 3:** Factors associated with fetal malnutrition at birth in South Gondar zone hospitals, northwest Ethiopia, 2020.

Variables	Fetal malnutrition		COR (95% CI)	AOR (95% CI)
	No	Yes		
Interpregnancy interval				
<24 months	260	68	0.93 (0.69-1.25)	
≥24 months	986	278	1	
Maternal height				
<150 centimeters	208	47	0.78 (0.56-0.11)	
≥150 centimeters	1038	299	1	
Newborn weight				
<2500 grams	230	101	1.82 (1.39-2.39)^∗^	1.33 (0.12-1.82)
≥2500 grams	1016	245	1	1
Intimate partner violence				
Yes	388	181	2.43 (1.90-3.09)^∗^	1.97 (1.52-2.56)^∗^
No	858	165	1	1
Gestational age				
Appropriate for gestational age	1032	232	1	1
Small for gestational age	214	114	2.37 (1.81-3.10)^∗^	1.96 (1.46-2.62)^∗^
Placental weight				
<512 grams	401	208	3.12 (2.48-4.06)^∗^	2.76 (2.13-3.57)^∗^
≥512grams	845	138	1	1
Altitude				
<2000 meters	352	74	1	1
2000-3000 meters	243	55	1.07 (0.73-1.59)	1.02 (0.67-1.55)
>3000 meters	651	217	1.59 (1.18-2.21)^∗^	1.24 (0.58-2.01)

NB. ^∗^Significant at *p* value < 0.05. COR = crude odds ratio; AOR = adjusted odds ratio; CI = confidence interval.

## Data Availability

The minimal dataset analyzed during the current study is available from the corresponding author on reasonable request.

## Abbreviations

AGA: Appropriate for gestational age
BMI: Body mass index
BP: Blood pressure
CAN: Clinical assessment of nutritional status
cm: Centimeter
EDHS: Ethiopian Demographic and Health Survey
FM: Fetal malnutrition
kg: Kilogram
m: Meter
MUAC: Mid upper arm circumference
IUGR: Intrauterine growth restriction/restriction
OR: Odds ratio
PI: Pondera index
SGA: Small for gestational age.
